# Genetic regulation of the placental transcriptome underlies birth weight and risk of childhood obesity

**DOI:** 10.1371/journal.pgen.1007799

**Published:** 2018-12-31

**Authors:** Shouneng Peng, Maya A. Deyssenroth, Antonio F. Di Narzo, Haoxiang Cheng, Zhongyang Zhang, Luca Lambertini, Arno Ruusalepp, Jason C. Kovacic, Johan L. M. Bjorkegren, Carmen J. Marsit, Jia Chen, Ke Hao

**Affiliations:** 1 Department of Genetics and Genomic Sciences, Icahn School of Medicine at Mount Sinai, New York, New York, United States of America; 2 Icahn Institute of Genomics and Multiscale Biology, Icahn School of Medicine at Mount Sinai, New York, New York, United States of America; 3 Department of Environmental Medicine and Public Health, Icahn School of Medicine at Mount Sinai, New York, New York, United States of America; 4 Department of Obstetrics, Gynecology and Reproductive Science, Icahn School of Medicine at Mount Sinai, New York, New York, United States of America; 5 Department of Cardiac Surgery, Tartu University Hospital, Tartu, Estonia; 6 Cardiovascular Research Centre, Icahn School of Medicine at Mount Sinai, New York, New York, United States of America; 7 Integrated Cardio Metabolic Centre, Department of Medicine, Karolinska Institutet, Karolinska Universitetssjukhuset, Huddinge, Sweden; 8 Environmental Health at Rollins School of Public Health, Emory University, Atlanta, Georgia, United States of America; 9 Department of Pediatrics, Icahn School of Medicine at Mount Sinai, New York, New York, United States of America; 10 Department of Oncological Sciences, Icahn School of Medicine at Mount Sinai, New York, New York, United States of America; 11 Department of Hematology and Medical Oncology, Icahn School of Medicine at Mount Sinai, New York, New York, United States of America; 12 Department of Respiratory Medicine, Shanghai Tenth People’s Hospital, Tongji University, Shanghai, China; 13 College of Environmental Science and Engineering, Tongji University, Shanghai, China; University of Chicago, UNITED STATES

## Abstract

GWAS identified variants associated with birth weight (BW), childhood obesity (CO) and childhood BMI (CBMI), and placenta is a critical organ for fetal development and postnatal health. We examined the role of placental transcriptome and eQTLs in mediating the genetic causes for BW, CO and CBMI, and applied integrative analysis (Colocalization and MetaXcan). GWAS loci associated with BW, CO, and CBMI were substantially enriched for placenta eQTLs (6.76, 4.83 and 2.26 folds, respectively). Importantly, compared to eQTLs of adult tissues, only placental eQTLs contribute significantly to both anthropometry outcomes at birth (BW) and childhood phenotypes (CO/CBMI). Eight, six and one transcripts colocalized with BW, CO and CBMI risk loci, respectively. Our study reveals that placental transcription *in utero* likely plays a key role in determining postnatal body size, and as such may hold new possibilities for therapeutic interventions to prevent childhood obesity.

## Author summary

Genetic studies (e.g GWAS) revealed substantial heritability on birth weight (BW), childhood obesity (CO) and childhood body mass index (CBMI), however, the etiological mechanisms and relevant tissue(s) underlying these traits/conditions are not clear. We incorporated the data from largest GWASes to date and placenta expressional quantitative trait loci (eQTL) that have been newly published, and showed the variants associated with BW, CO and CBMI were substantially enriched for placenta eQTLs (6.76, 4.83 and 2.26 folds, respectively). Importantly, compared to eQTLs in 7 adult tissues such as adipose and liver, only eQTLs in the placenta were found to contribute significantly not only to anthropometry outcomes at birth (BW) but also to childhood phenotypes (CO/CBMI). Further, we employed COLOC and MetaXcan analyses and identified placenta transcripts potential mediate the genetic effect of BW/CO/CBMI GWAS loci. In summary, our study strongly supports a key role for the placenta in determining BW, CO and CMBI at the molecular level, and pinpointed genes whose expression levels in placenta potentially influences BW and CO risk.

## Introduction

Birth weight (BW) is influenced by both fetal and maternal factors, including race, infant sex, plurality, altitude, education, and smoking [1], and is consistently associated with future risk of adult metabolic diseases including type 2 diabetes (T2D) and cardiovascular disease [2]. Childhood obesity (CO) has also emerged as an important health problem in the United States and most other countries in the world [3,4]. In the USA, 1 in 3 children is afflicted with overweight or obesity [3]. The increasing prevalence of CO is also associated with diseases later in life including T2D, hypertension, nonalcoholic fatty liver disease, obstructive sleep apnea, and dyslipidemia [3].

The placenta, situated at the maternal-fetal interface, is a key organ for fetal growth and development; it performs a variety of functions including controlling fetal access to nutrients, hormone production, and mitigation of adverse effects from the environment [2,5,6]. The placenta and associated extraembryonic membranes are of fetal organ (i.e, have the same genetic composition as the fetus), formed from the zygote at the start of each pregnancy [7]. Several studies, including our own, have reported associations linking placenta markers to variation in birth weight [8,9]. Recently, we leveraged the Rhode Island Child Health Study (RICHS) [6,10] resource and reported the first placenta expression quantitative trait loci (eQTL) profile based on a large sample size [11]. We discovered more than 3,000 *cis-* and *trans*-eQTLs at ≤10% false discovery rate (FDR), demonstrating that the placenta is a transcriptionally active tissue and that this activity is largely controlled by fetal genetics [11]. Large meta-analysis of BW [12], CO [13] and childhood BMI (CBMI) [14] genome-wide association studies (GWAS) have identified multiple genome-wide significant loci suggesting a strong genetic determinant for these conditions. At genome-wide significance, 60 and 15 fetal loci have been associated with BW (explaining 15% of its variance)[12] and CBMI[14], respectively. As many of these genome-wide significant variants are in non-coding regions, they likely affect these phenotypes through regulation of gene expression. Furthermore, while genetic variants are static within an individual, the regulatory effect that a genetic polymorphism exerts on outcomes will be likely to vary spatially, temporally and in response to differing environmental exposures and influences. As a noted limitation of the GWAS design, while informative for identifying genetic loci implicated in disease, this approach does not provide information on down-stream genes and their pathophysiological functions. More specifically, the GWAS design is unable to identify; (1) the tissue types and developmental stages where the causal variants and the underlying genes have their effects and (2) the mechanisms of effect of a causal variant.

An eQTL analysis provides a profile of significant associations between transcription levels of a gene and genetic polymorphisms. A related concept, eSNP, denotes a particular SNP whose genotype is significantly associated with the transcription level of a gene. A distinction is made as to the location of the eQTL relative to the physical location of the gene whose expression level it is associated with; if the eQTL is close (within 500kb) to the gene (which encodes the mRNA), it is defined as a cis-eQTL. In contrast, if the eQTL is distant (typically more than 500kb away or on another chromosome altogether) from the gene whose expression level it is associated with, it is defined as a trans-eQTL. In this study, the cis- and trans-eQTLs in placenta and adult tissues were defined using these criteria, as previously reported [11]. An important approach is to apply knowledge of eQTLs in disease-relevant tissues to mine GWAS risk loci datasets to discover underlying mechanisms linking genetic variants to diseases [15,16]. Recently, using integrative genomics applied to eQTLs has become an even more powerful tool to mine GWAS results and shed light on disease pathways, as it can identify true disease causal variants (as opposed to merely undirected associations) with down-stream genes in a tissue-specific manner. Recently, methods like COLOC [17], SMR [18], MetaXcan [19], and TWAS [20] have shown superior ability to identify causal genes of genetic risk loci compared to earlier integrative analysis simply looking at overlap between eQTLs and GWAS lead SNPs.

Herein, to study the *in utero* genes/pathways influencing BW, CO and CBMI, and to define the specific role of the placenta for neonatal and long-term child health outcomes, we compared placenta eQTLs with 7 STARNET adult tissue eQTLs (blood, atherosclerotic-lesion-free internal mammary artery, atherosclerotic aortic root, subcutaneous fat, visceral abdominal fat, skeletal muscle, and liver). Further, we applied COLOC and MetaXcan analyses on placenta eQTLs integrated with GWAS data.

## Results

### GWAS loci for birth weight, childhood BMI and childhood obesity are enriched for placenta eQTLs

**Fig 1:**
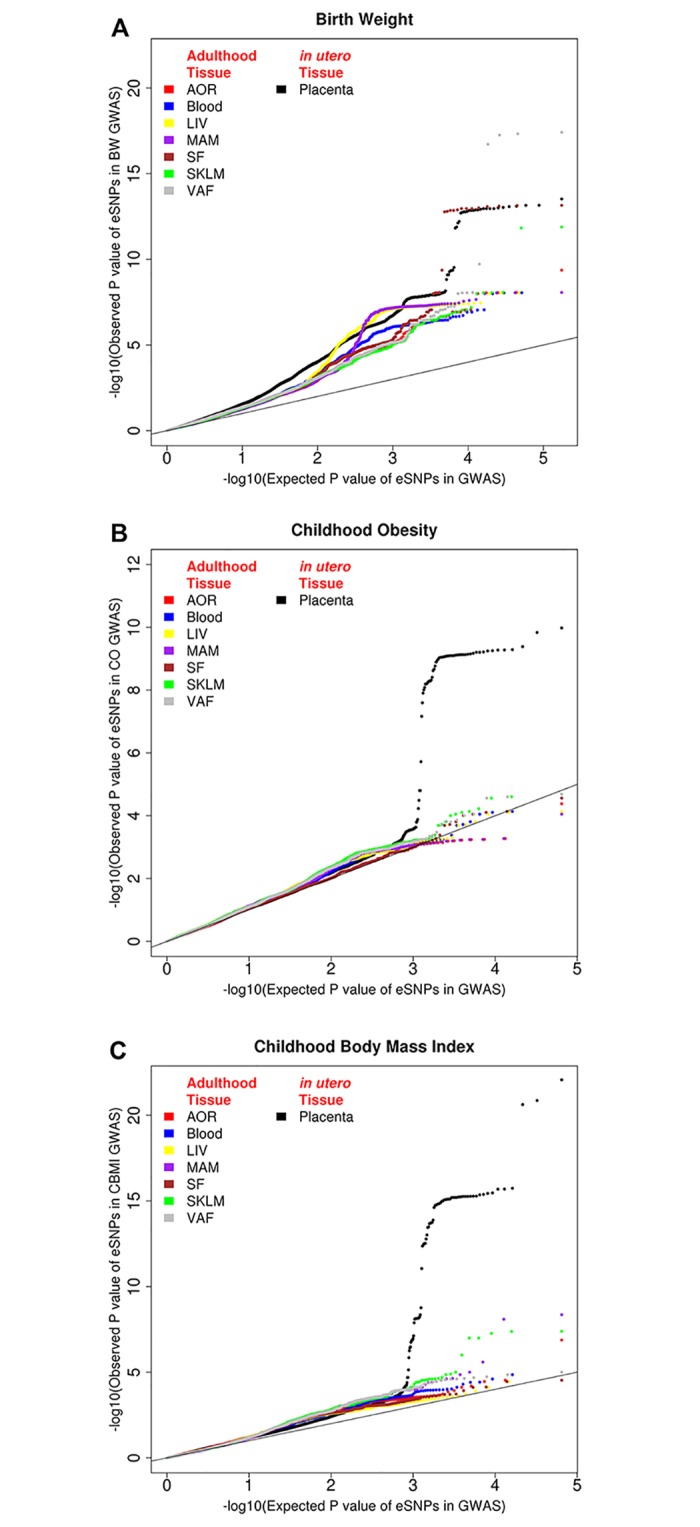
Enrichment of small pvalues for RICHS placenta tissue eSNPs and for eSNPs of adult tissues (STARNET cohort, Materials and methods) in A, birth weight (BW), B, childhood obesity (CO), and C, childhood body mass index (CBMI) GWASes. Adult tissues: blood, atherosclerotic-lesion-free internal mammary artery (MAM), atherosclerotic aortic root (AOR), subcutaneous fat (SF), visceral abdominal fat (VAF), skeletal muscle (SKLM), and liver (LIV).

Summary level data of three GWAS meta-analyses were retrieved (Materials and methods): (1) a multi-ancestry GWAS meta-analysis of BW in 153,781 individuals [12]; (2) a CBMI GWAS meta-analysis of 20 studies with a sample size of 35,668 [14], where the BMI was measured between 2 and 10 years of age; (3) a CO meta-analysis of 14 studies consisting of 5,530 cases and 8,318 controls [13], where cases were defined as BMI>95th percentile and controls defined as BMI<50th percentile. We overlapped the GWAS results (summary level data, Materials and methods) of BW, CO and CBMI with placenta eSNPs (10% FDR), and found that placenta eSNPs were substantially enriched for GWAS hits with highly significant p-values (Fig 1). Among GWAS hits with p-value ≤ 10^−5^, placenta eSNPs were 6.76 fold enriched for BW, 4.83 fold for CO; and 2.26 for CBMI (S1 Table). We explored similar GWAS-eQTL overlapping in tissue types collected in adult subjects using eSNPs (FDR≤10%) from the STARNET study[21] (Materials and methods). All tissues’ eSNPs were enriched for BW GWAS signals, and the enrichment were significant (pvalue<1e-6) in Kolmogorov—Smirnov test (K-S test) even after LD pruning (Materials and methods). The eSNPs in subcutaneous fat and visceral abdominal fat tissues demonstrated most substantial enrichment among adult tissues eQTLs, comparable to that of placenta eSNPs (Fig 1A). Interestingly, unlike the placenta, none of the adult tissue eQTLs in STARNET demonstrated magnitude of enrichment in CO or CBMI GWAS hits comparable to placenta eSNPs (Fig 1B and 1C), indicating a more prominent role of the placenta transcriptome in postnatal anthropometry outcomes.

### Colocalization of genetic control of placenta transcriptome with BW, CO and CBMI

We integrated placenta eQTLs with GWAS summary data using the COLOC approach [17] to identify transcripts influenced by the same SNPs driving disease phenotype. COLOC was conducted on 1,863 non-overlapping intervals around BW GWAS hits (Materials and methods), and eight transcripts (*ITPR2*, *PLEKHA1*, *TBX20*, *METTL21A*, *SPATA20*, *TMEM87B*, *RPS9*, and *HAUS4*) were identified as PP.H_4_ >75% (Table 1). The finding suggested, at these loci, a single genetic variant controlled both BW and transcript levels in placenta. Six genes were genetically co-localized with CO [13] with PP.H_4_ >75% and one gene was co-localized with CBMI (Table 1). All transcripts identified by COLOC analysis were protein coding genes. We also conducted COLOC integrating placenta eQTLs and adult BMI [22], and no transcript showed large PP.H_4_ (e.g. ≥0.75).

**Table 1:** Genetic colocalization of placenta eQTLs with birth weight, childhood obesity and childhood BMI GWAS loci.

Trait	Gene ID	Gene Symbol	COLOC Posterior Probability^†^				
			H_0_	H_1_	H_2_	H_3_	H_4_
**Birth Weight**							
	ENSG00000123104	ITPR2*	2.3E-07	1.2E-04	3.8E-05	1.9E-02	9.8E-01
	ENSG00000107679	PLEKHA1*	3.2E-19	2.9E-04	3.3E-17	2.8E-02	9.7E-01
	ENSG00000164532	TBX20*	1.5E-29	4.1E-05	2.8E-26	7.7E-02	9.2E-01
	ENSG00000144401	METTL21A*	5.7E-06	7.8E-03	5.9E-05	8.0E-02	9.1E-01
	ENSG00000006282	SPATA20*	8.7E-44	3.9E-02	1.1E-43	5.1E-02	9.1E-01
	ENSG00000153214	TMEM87B*	3.4E-03	2.2E-02	1.1E-02	6.7E-02	9.0E-01
	ENSG00000170889	RPS9	1.5E-04	6.1E-02	1.3E-04	5.3E-02	8.8E-01
	ENSG00000092036	HAUS4*	1.9E-04	6.6E-02	3.1E-04	1.0E-01	8.3E-01
**Childhood Obesity**							
	ENSG00000213214	ARHGEF35	8.2E-03	3.4E-02	3.4E-03	1.3E-02	9.4E-01
	ENSG00000085063	CD59	7.6E-03	6.6E-02	1.3E-03	1.0E-02	9.1E-01
	ENSG00000138031	ADCY3*	1.2E-10	8.7E-07	1.4E-05	9.5E-02	9.0E-01
	ENSG00000198793	MTOR	3.1E-02	1.5E-02	1.1E-01	5.1E-02	8.0E-01
	ENSG00000150403	TMCO3	3.8E-03	1.8E-01	9.7E-04	4.4E-02	7.7E-01
	ENSG00000010030	ETV7	2.6E-24	2.2E-01	1.3E-25	1.1E-02	7.7E-01
**Childhood BMI**							
	ENSG00000165238	WNK2	4.5E-03	4.5E-03	9.6E-02	9.5E-02	8.0E-01

* These genes were also identified in MetaXcan analysis (Table 2);

^†^ Genes of COLOC Posterior Probability of H_4_≥ 0.75 were presented

### Association of imputed placenta transcription level with BW, CO and CBMI

We implemented MetaXcan [19] to integrate GWAS summary data (BW, CO and CBMI) and sample level placenta eQTL data to identify genes whose imputed expression level was associated with GWAS traits. Table 2 lists the findings that passed Bonferroni correction (Materials and methods). Nineteen genes were associated with BW. Importantly, seven out eight genes genetically colocalized with BW were also identified by MetaXcan (Table 2). Further, MetaXcan inferred the directionality of the transcription-trait association, for example, higher *PLEKHA1* mRNA level in placenta was associated with low birth weight. The expression level of three genes (*ADCY3*, *DNAJC27*, *TYW3*) were associated with childhood obesity risk. *ADCY3* and *DNAJC27* belong to the genomic locus, and both have positive correlations with CO and CBMI (Table 2).

**Table 2:** Imputed gene expression levels associated^†^ with birth weight, childhood obesity and childhood BMI.

Gene ID	Gene Symbol	MetaXcan		Gene ID	Gene Symbol	MetaXcan	
		Effect Size	P value			Effect Size	P value
**Birth Weight**				**Childhood Obesity**			
ENSG00000181885	CLDN7	0.0754	6.78E-15	ENSG00000138031	ADCY3	0.4200	2.62E-10
ENSG00000164532	TBX20	-0.0247	8.38E-09	ENSG00000115137	DNAJC27	0.4494	1.69E-05
ENSG00000123104	ITPR2	-0.0519	7.80E-07	ENSG00000162623	TYW3	-1.319	8.34E-04
ENSG00000144401	METTL21A	0.0396	1.37E-06				
ENSG00000114127	XRN1	0.0688	1.40E-06	**Childhood BMI**			
ENSG00000170606	HSPA4	-0.0773	8.31E-06	ENSG00000138031	ADCY3	0.1755	6.00E-17
ENSG00000107679	PLEKHA1	-0.0253	9.22E-06	ENSG00000135451	TROAP	-0.5336	2.78E-10
ENSG00000153214	TMEM87B	0.0646	9.82E-06	ENSG00000134287	ARF3	-0.1280	3.91E-08
ENSG00000092036	HAUS4	0.0539	1.22E-05	ENSG00000115137	DNAJC27	0.1537	1.11E-06
ENSG00000144891	AGTR1	0.1049	1.32E-05	ENSG00000001617	SEMA3F	0.2385	1.63E-04
ENSG00000090621	PABPC4	0.0483	1.68E-05	ENSG00000138615	CILP	-0.1585	2.32E-04
ENSG00000128891	C15orf57	-0.0214	3.07E-05				
ENSG00000146374	RSPO3	0.0576	3.31E-05				
ENSG00000006282	SPATA20	-0.0158	3.57E-05				
ENSG00000141295	SCRN2	0.0228	4.50E-05				
ENSG00000124467	PSG8	0.0343	7.29E-05				
ENSG00000159111	MRPL10	-0.0655	7.35E-05				
ENSG00000166033	HTRA1	-0.0289	8.24E-05				
ENSG00000172243	CLEC7A	-0.0617	8.97E-05				

^†^Only MetaXcan results that passed Bonferroni correction are shown. The Effect Size refers to the change of gene expression associated with phenotype trait (ie, birth weight, childhood obesity, and childhood BMI) inferred by MetaXcan. Positive effect size indicates the gene expression level is positively correlated with the phenotypic trait; and negative effect size indicates negative correlation.

### Direct validation of association between BW and measured transcription levels in RICHS

We corroborated the MetaXcan-based inferred directionality of the imputed transcription vs. BW associations using available data: observed BW and placenta transcription (whole transcriptome RNAseq, Materials and methods) in the RICHS cohort. Out of the 19 genes identified by MetaXcan (Table 2), placenta mRNA level of seven genes was associated with BW with FDR<0.1, including *XRN1*, *HSPA4*, *PLEKHA1*, *AGTR1*, *RSPO3*, *MRPL10*, and *CLDN7* (S2 Table). Importantly, among these seven genes, the direction of six genes (except *CLDN7*) association with BW were consistent with that inferred by MetaXcan. The association between observed BW and measured expression level of these seven genes were robust even we adjusted the regression model for gestational age, baby sex, race, peak eSNP and GWAS peak SNP.

### Correlation among BW and CO, CBMI

Based on the GWAS summary level data on BW, CO and CBMI, we applied LD score regression [23,24] and found relatively high heritability (h^2^) of BW, CO and CBMI, being 0.1023, 0.4067, and 0.2421, respectively (Table 3). The heritability of CO is particularly large compared to other heritable traits. For example, h^2^ of T2D is 0.0872 and h^2^ of BMI is 0.1855[24]. In fact, the heritability of CO is comparable to that of height (h^2^ = 0.4623) [24,25], indicating it is strongly controlled by genetic background. Birth weight has significant genetic correlation (r_g_) with CO and CBMI at 0.1847 and 0.2038, respectively (Table 3), indicating BW and CO/CMBI share genetic determinants. Importantly, the positive r_g_ demonstrates that higher BW is genetically associated with higher CBMI value and increased CO risk. We identified the “shared SNPs” that were associated with both BW and CO at GWAS p-value cutoffs of 1e-3, 1e-2 and 1e-1 (Table 4). Consistent with the positive r_g_, most shared SNPs have consistent allele direction in BW and CO (Table 4). It should be noted that few “shared SNPs” showed highly significant (e.g. p value <1e-4) association with both traits, and in fact, most of the shared SNPs had moderate significance (e.g. 1e-2), indicating that the common genetic predisposition of BW and CO is not attributable to a small number of loci of large effects, but rather to many genetic variants of small-to-moderate effect size.

**Table 3:** Heritability and genetic correlation among birth weight, childhood obesity and childhood BMI*.

	Heritability		Genetic Correlation with BW	
	H^2^ (se)	P value	r_g_ (se)	P value
**Birth Weight**	0.1023(0.0065)	8.2E-56	-	-
**Childhood Obesity**	0.4067(0.0423)	6.9E-22	0.1847(0.0482)	1.0E-04
**Childhood BMI**	0.2421(0.0221)	6.3E-28	0.2038(0.0432)	2.4E-06

* H^2^, heritability; r_g_, genetic correlation; se, standard errors.

**Table 4:** Overlap of GWAS signals for birth weight GWAS and childhood obesity GWAS.

GWAS P value threshold	Overlap OR	Number of overlap SNPs	N of overlap SNPs consistent risk allele	% SNPs with consistent risk allele	p-value of risk allele consistent
1.0E-03	1.55	36	33	91	3.52E-07
1.0E-02	1.77	1062	713	67	7.07E-29
1.0E-01	1.12	32613	20336	62	1.53E-322

P value threshold; GWAS p value threshold applied to both birth weight GWAS and childhood obesity GWAS. Consistent direction allele; the SNPs where the same allele association with higher birth weight and higher child obesity.

## Discussion

There is increasing recognition that the period of intrauterine development constitutes one of the most critical periods for defining disease risk later in life [26]. We report the first study to use comprehensive transcriptome data from the placenta (being one of the most relevant tissues in controlling *in utero* development) to interpret the causal effects of GWAS genetic risk loci for BW, CO and CBMI. We found extensive enrichment of placenta eQTLs for GWAS hits (Fig 1), and importantly, discovered hundreds of transcripts controlled by the genetic variants that influence these anthropometric traits. Our findings demonstrate that placental transcription and related functions defined by identified trait genes play important roles in determining BW, CO and CBMI

BW, CO and CBMI are of major clinical and public health importance. BW has been convincingly shown to be inversely associated with risk for T2D, CAD and hypertension in later life [12]. CO or excess BMI are risk factors for T2D and hypertension [3]. These traits are also heritable, especially CO (h^2^ = 0.4067). Furthermore, these traits are genetically correlated (Table 3), where higher BW is associated with increased risk of CO. Importantly, we demonstrate that the placenta is a highly relevant tissue for determining not only weight at birth (BW) but also body weight in childhood (CO and CBMI). Identifying the genes mediating the genetic predisposition for BW, CO and CBMI is of great importance for understanding the mechanisms controlling these traits and developing clinical prevention strategies.

Integrative genomics can be a powerful tool to mine GWAS results. Many methods have been proposed to integrate GWAS summary data and eQTL data, where GWAS and eQTL studies used different datasets or population. These methods include SMR, PrediXcan, Sherlock, COLOC, eCAVIAR, etc, and they can be categorized into two broad classes [27]. (1) Class 1 includes TWAS, MetaXcan and SMR, which are tests for significant genetic correlation between cis expression and GWAS. (2) Class 2 includes COLOC and eCAVIAR, which are estimations (rather than tests) of the posterior probability of colocalization, where colocalization is defined as shared causal variant(s) between the expression and GWAS. It has been reported that the results of these two classes of methods do not fully overlap [28]. Possible explanations are different assumption, algorithm, power, etc. Herein, we took a common strategy [28] and report all gene pinpoint by COLOC and/or MetaXcan, as they are potentially on the causal pathway of BW, CO and CBMI. In S3 Table, we report the LD between the lead eQTL and the lead GWAS SNP near the gene locus.

**Fig 2:**
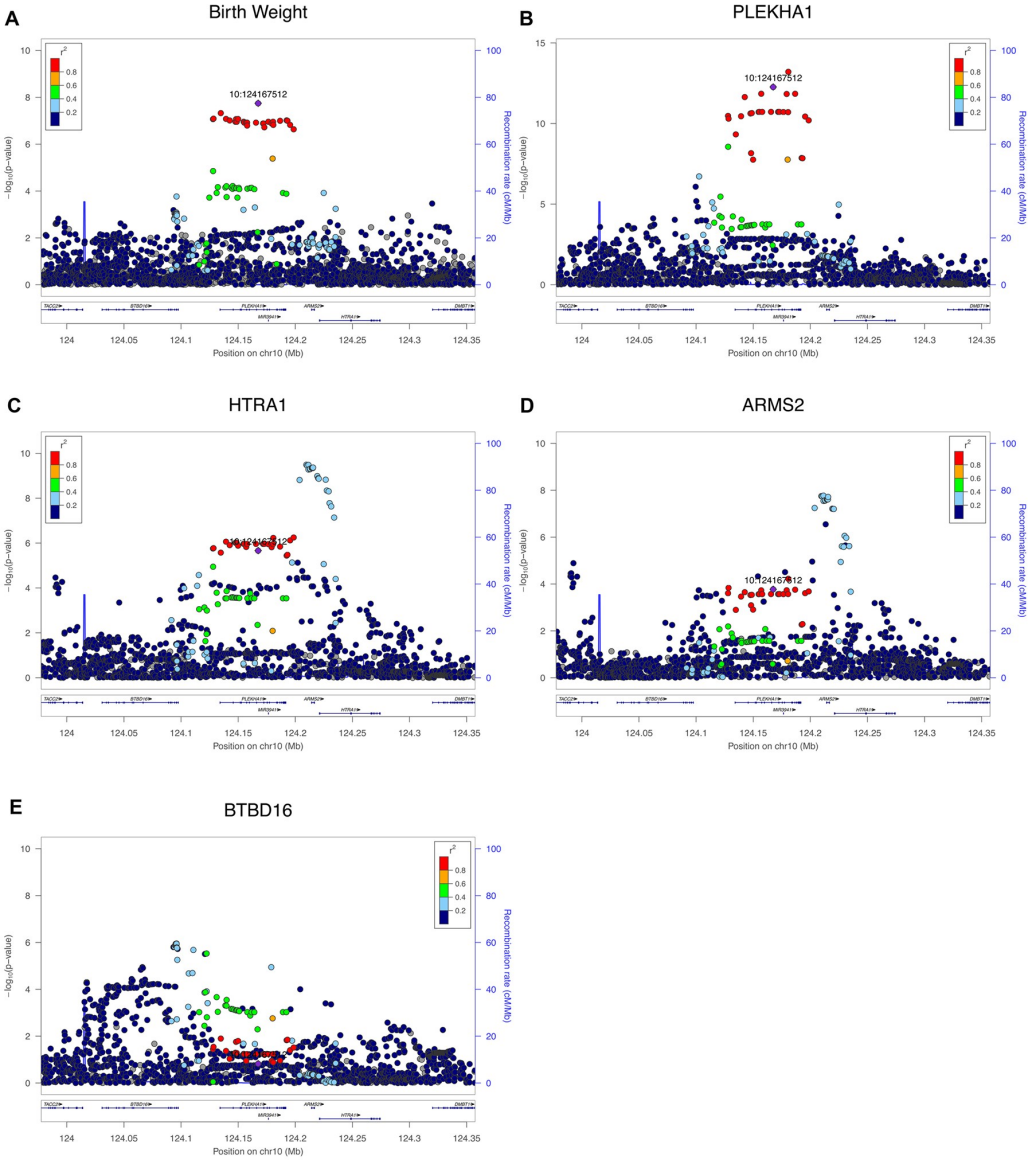
A, LocusZoom plots of BW GWAS peak with lead SNP rs2421016 (chr10:124167512). B, C, D, E, LocusZoom plots of eQTL peaks on *PLEKHA1*, *HTRA1*, *ARMS2*, *BTBD16* genes, respectively. In these plots, each dot denotes a variant. Purple dots: lead SNP chr10:124167512; red and orange dots, SNPs of strong LD (r^2^>0.6) with lead SNP; green and light blue dots, SNPs of moderate LD (0.6>r^2^>0.2) with the lead SNP; and dark blue dots, SNPs of weak or no LD (r^2^<0.2) with the lead SNP.

There are six genes coded by a single BW GWAS locus with lead SNP rs2421016 (chr10:124167512) (Fig 2A), and we found that four out of the six genes were co-localized with BW (Table 1): *PLEKHA1* (Pleckstrin Homology Domain Containing A1), *HTRA1* (HtrA Serine Peptidase 1), *ARMS2* (Age-Related Maculopathy Susceptibility 2), and *BTBD16* (BTB Domain Containing 16). The eQTLs of these four genes also overlap with BW GWAS peaks (Fig 2B–2E). In particular, the shape of the *PLEKHA1* eQTL overlaps well with the BW GWAS peak (Fig 2A and 2B), where the top eSNPs include the GWAS lead SNP (purple dots) and the SNPs in strong LD with the lead SNPs (red and orange dots). In COLOC analysis, *PLEKHA1* showed PP.H_4_ much larger than PP.H_3_, indicating BW and *PLEKHA1* expression levels share a single causal SNP. For *HTRA1*, *ARMS2* and *BTBD16* genes, the GWAS lead SNP and its high LD proxy SNPs (purple, red and orange dots) only showed association with transcription at moderate significance, while the top eSNPs are SNPs with moderate LD with the GWAS lead SNP (green and light blue dots), suggesting multiple SNPs influence *HTRA1*, *ARMS2* and *BTBD16* expression levels and some of these SNPs also impact BW. Of relevance, a study in a murine model found *Htra1* is associated with placenta development [29]. In humans, upregulation of *ARMS2* was observed in placenta of growth-restricted infants [29], and perturbations in *ARMS2* may result in dysfunction of the extracellular matrix; suggesting that up-regulation of *ARMS2* forms part of an important survival mechanism to compensate for placental growth discordance [29]. Furthermore, various GWAS have shown this locus (10q26.13) is associated with multiple diseases/traits, including age-related macular degeneration [30], type 2 diabetes [31,32], migraine [33] and height [34], suggesting that the versatile functions of these genes may affect many biological processes.

In this paper, we report BW, CO, and CBMI GWAS signals are more profoundly enriched in placenta eQTLs comparing to adult tissue eQTLs (ie. STARNET). The differences are not driven by a few loci but rather polygenic. After strict LD pruning (Materials and methods), which eliminated the eSNPs that are in high LD, the conclusions remain unchanged that GWAS signals are more profoundly enriched in eQTLs of placenta than adult tissues (S1 Fig). Two-sample K-S test (on pruned SNP lists) showed such difference in enrichment magnitude were statistically significant (S4 Table). Such results suggest placenta could be a relevant tissue type controlling these neonatal traits. As a future direction, it would be important to investigate whether eQTLs of infant or pediatric tissues (e.g. skeleton muscle) substantially enrich for BW, CO, CBMI GWAS loci and compare to the results of placenta.

The developmental origins of health and disease (DOHaD) hypothesis emphasizes the role of the *in utero* environment on shaping the developmental trajectory of the fetus and, thereby, subsequent health outcomes across the lifespan. Our findings showcasing the influence of genetically controlled *in utero* expression patterns linked to BW, CO and CBMI also support this hypothesis. One of our key observations is that placental eQTLs are enriched to a greater extent for CO/CBMI GWAS hits than eQTLs from other tissues, including adipose and liver (Fig 1B and 1C). This observation strongly suggests that the genomic control and transcription activity of these genes in the placenta has a profound and long lasting impact on postnatal development and human health. In addition, the transcripts potentially mediating the genetic control of BW and CO can be influenced by environmental factors, and our results shed light on environmental health and nutritional elements that are likely involved in the childhood obesity epidemic [4]. Moreover, our analysis also revealed the genes/transcripts likely to mediate the effects of genetic polymorphisms on BW and CO/CBMI, as well as the directionality of the mediation effect; thus providing prioritized targets for experimental follow-up, intervention and prevention in the fight against the current obesity epidemic.

## Materials and methods

### Ethics statement

All GWAS summary level data are available from third party sources and no additional ethics approval is needed. The RICHS eQTL data is available in supplementary files, and the study protocol was reviewed and approved by the Office of Human Research Protections registered Institutional Review Boards [10].

### Birth weight GWAS summary level data

We retrieved summary level data of a genome-wide association study (GWAS) meta-analysis of birth weight in 153,781 individuals, where multiple births were excluded. Further, extreme values (< 2.5 kg or > 4.5 kg) were excluded as implausible for live term births before 1970 [12]. Only GWAS meta-analysis summary level data of the Caucasian part of study (N = 143,677) is used in this paper.

### Childhood BMI and childhood obesity GWAS summary level data

We retrieved summary level data from the Early Growth Genetics Consortium. In the Childhood obesity meta-analysis of childhood BMI in subjects of European ancestry, the BMI was measured between 2 and 10 years. In case of multiple births (twins, triplets), only one child was included. The GWAS meta-analysis included 20 studies with a sample size of 35,668 [14]. In Childhood obesity meta-analysis of childhood obesity in subjects of European ancestry, cases were defined as BMI>95th percentile; and controls defined as BMI<50th percentile. The meta-analysis was performed on 14 studies consisting of 5,530 and 8,318 controls [13].

### Adult BMI GWAS summary level date

We retrieved summary level data on adult BMI trait from the GIANT Consortium [22]. This adult BMI study is GWAS meta-analysis of 82 cohorts of 236,231 subjects in total. The summary level data was used in COLOC analysis and compared to the results of childhood BMI.

### Rhode Island child health study and placenta eQTLs

The Rhode Island Child Health Study (RICHS)[10] consists of singleton, term infants (≥37 weeks gestation) born without serious pregnancy complications or congenital or chromosomal abnormalities. Birth weight of the RICHS subjects (newborns) was recorded and placenta tissue collected for RNA and DNA extraction [11]. Four full thickness biopsies free of maternal decidua were taken, one from each of four quadrants around the placenta, within 2 cm of the cord insertion site, and immediately placed in RNALater preservative. After at least 24 hours, these samples were snap frozen in liquid nitrogen and homogenized together into a powder, and aliquots of that homogenized sample were used with the intent to reduce variation based on location. RNA and DNA were profiled using whole transcriptome RNAseq and Illumina Mega^EX^ SNP array, respectively [11]. After stringent QC, a subset of the RICHS placenta collections (N = 150) with high quality RNAseq and whole-genome genotyping data were used for generating eQTLs. The data is primarily of Caucasian ancestry (77.3%) and the race composition is presented in S5 Table. Placenta eQTLs were previously reported and are publically available [11].

### Stockholm-Tartu atherosclerosis reverse networks engineering task study eQTLs

We compared placenta tissue eQTLs to those from adult tissues in the Stockholm-Tartu Atherosclerosis Reverse Networks Engineering Task study (STARNET) [21]. The genotyping and RNAseq of RICHS placenta and STARNET samples were conducted under similar conditions. We surveyed seven tissues: blood, atherosclerotic-lesion-free internal mammary artery (MAM), atherosclerotic aortic root (AOR), subcutaneous fat (SF), visceral abdominal fat (VAF), skeletal muscle (SKLM), and liver (LIV). We randomly selected 150 samples from each STARNET tissues (the same sample size as RICHS placenta set) and computed 10% FDR eQTLs for integration with BW, CO and CBMI GWAS data.

### Evaluation of significance level for eSNPs enrichment in GWAS

We are interested in evaluating the significance level of eSNPs’ enrichment for small GWAS pvalues, which is visualized in Fig 1. We (1) identified the shared SNPs that are in the GWAS study (e.g. BW GWAS) and are also eSNPs (≤10% FDR) in the tissue of interest; (2) conducted LD pruning on the SNP list using a rather stringent threshold (ie, r^2^≤0.2), which capped the r^2^ among the pruned SNPs at 0.2; (3) performed Kolmogorov-Smirnov test (K-S test) between the pvalues of pruned SNPs and null distribution. Similar approach was applied to compare magnitude of enrichment between eSNPs of two tissues, where we performed two-sample K-S test on the pruned SNP lists derived from the two tissues (S4 Table).

### Colocalization of GWAS peak SNPs and placenta eSNPs

We firstly define “intervals” around GWAS association signals. GWAS SNP of pvalue <1e-3 were selected, and an interval is formed around each SNP by extending 200kb both upstream and downstream. Then we merge overlapping intervals into larger intervals until the remaining intervals no longer overlap. Colocalization analysis was performed within each interval using COLOC version 2.3–6 in R[17]. This method assesses whether two association signals, GWAS summary statistics and eQTL statistics, are consistent with shared causal variant(s) [17]. Default priors of the software were used: p1, prior probability a SNP is associated with trait 1, default: 1e-4; p2, prior probability a SNP is associated with trait 2, default: 1e-4; p12, prior probability a SNP is associated with both traits, default: 1e-5. Intervals were created around each GWAS SNP (with GWAS pvalue<1e-3) ± 200Kb, and overlapping intervals were merged into one interval. Afterwards, COLOC analysis was conducted for each interval, where only eSNPs and transcripts located within the interval entered analysis. In total, five hypotheses were evaluated. H_0_: No association with either disease risk (i.e., trait 1) or placenta gene expression (i.e., trait 2); H_1_: Association with trait 1, not with trait 2; H_2_: Association with trait 2, not with trait 1; H_3_: Association with trait 1 and trait 2, multiple independent SNPs influencing the two traits; H_4_: Association with trait 1 and trait 2, one shared SNP. Genes that demonstrated a high posterior probability of hypothesis 4 (PP.H_4_ >75%) indicate the disease risk and placenta gene expression were controlled by the same genetic variant.

### LD score regression analysis

LD Score Regression [23] version 1.0.0 was employed to estimate the heritability and genetic correlation of BW, CO and CBMI traits. The European subjects in the 1000Genome data set were used as an LDscore reference.

### MetaXcan

We applied MetaXcan [19] to integrate GWAS summary level data and sample level placenta eQTL data (genotype and gene expression) to identify genes underlying BW, CO and CBMI traits. To reduce the multiple testing burden, we limited the test to genes influenced by placenta eSNPs (≤10% FDR), and with at least one of the genes’ eSNP having a pvalue ≤ 0.01 in the GWAS of interest. For BW, 487 genes were evaluated; for CO, 163 genes were evaluated; and for CBMI, 169 genes were evaluated. Bonferroni correction was applied to adjust pvalues.

### Association between observed BW and measured transcription level in RICHS data

We took advantage of the RICHS data which measured both birth weight and placenta gene expression levels in the same set of subjects, and validated significant MetaXcan findings (ie, association between BW and imputed placenta transcription levels). Briefly, generalized linear models (glm) were performed on transcripts potentially responsible in controlling BW (identified in MetaXan, Table 2).

## Supporting information

S1 Fig After LD pruning (max(r^2^)≤0.2), enrichment of small pvalues for RICHS placenta tissue eSNPs and for eSNPs of adult tissues (STARNET) in A, birth weight (BW), B, childhood obesity (CO), and C, childhood body mass index (CBMI) GWASes. Adult tissues: blood, atherosclerotic-lesion-free internal mammary artery (MAM), atherosclerotic aortic root (AOR), subcutaneous fat (SF), visceral abdominal fat (VAF), skeletal muscle (SKLM), and liver (LIV).
(TIF)

S1 Table Enrichment of placenta eQTLs among GWAS signals for birth weight, childhood obesity and childhood body mass index. (DOCX)

S2 Table Association between observed birth weight and measured gene expression in RICHS cohort. (XLSX)

S3 Table LD (r2) between the GWAS lead SNP and the eQTL lead SNP around each gene identified in COLOC and MetaXcan. (XLSX)

S4 Table Comparing the eSNPs enrichment for GWAS signals of placenta vs. adult tissues. (DOCX)

S5 Table Ethnicity distribution of the RICHSs placenta data set (n = 150). (DOCX)

S1 Data Placenta eQTLs summary statistics. The statistics include Beta, SE, Pvalue, gene_id, CHROM:POS:NON_EFF:EFF, and EAF. SE, standard error of beta; POS, the SNP co-ordinates based on hg19; NON_EFF, the non-effective allele; EFF, the effective allele; EAF, the effective allele frequency. The cis-window is defined as [TSS - 500kb, TES + 500kb], where TSS denotes transcription start site and TES denotes transcription end site.
(ZIP)

S2 Data Gene annotation information. (GZ)

S3 Data Md5sum for S1 and S2 Datas. (MD5)
