# Supplementary figures and images for: Genetic regulation of the placental transcriptome underlies birth weight and risk of childhood obesity
Source: PLoS Genet. 2018 Dec 31;14(12):e1007799. doi: 10.1371/journal.pgen.1007799 (PMC6329610; doi:10.1371/journal.pgen.1007799)

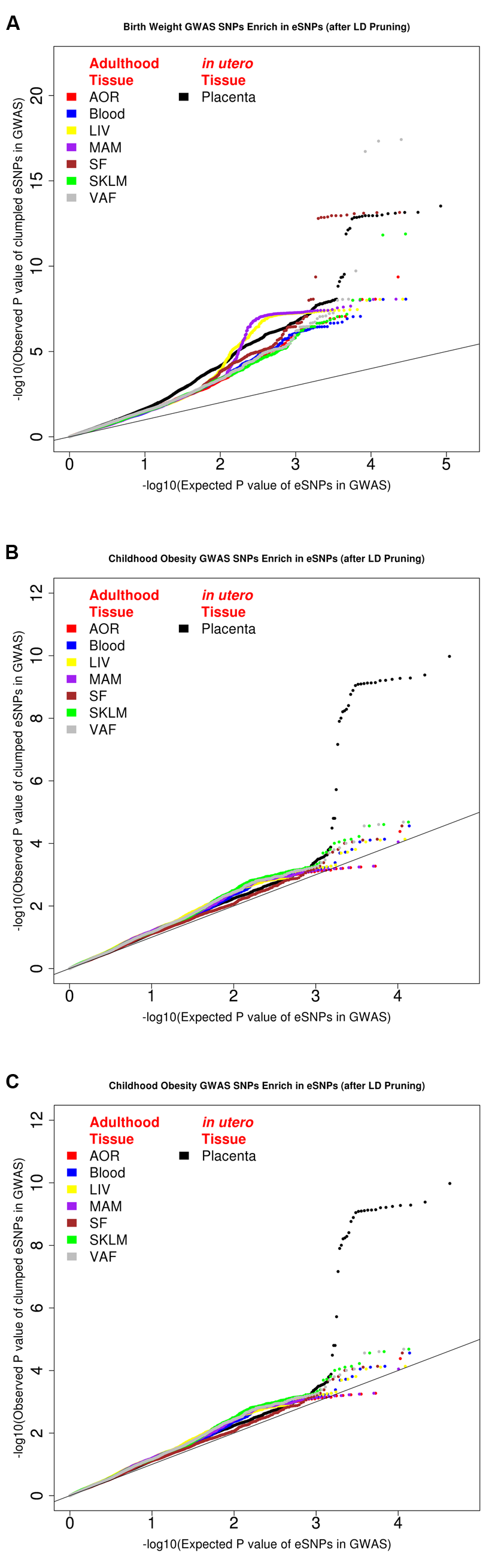

Supplement: S1 Fig — Adult tissues: blood, atherosclerotic-lesion-free internal mammary artery (MAM), atherosclerotic aortic root (AOR), subcutaneous fat (SF), visceral abdominal fat (VAF), skeletal muscle (SKLM), and liver (LIV). (TIF) [file pgen.1007799.s001.tif]
