# Supplementary material for: Genetic regulation of the placental transcriptome underlies birth weight and risk of childhood obesity
Source: PLoS Genet. 2018 Dec 31;14(12):e1007799. doi: 10.1371/journal.pgen.1007799 (PMC6329610; doi:10.1371/journal.pgen.1007799)
Supplement: S1 Table — (DOCX) [file pgen.1007799.s002.docx]

**Table. Enrichment of placenta eQTLs among GWAS signals for Birth Weight, Childhood Obesity and Childhood BMI**

| **GWAS Pvalue Cutoff** | **Birth Weight** | |  | **Childhood Obesity** | |  | **Childhood BMI** | |
| --- | --- | --- | --- | --- | --- | --- | --- | --- |
|  | # overlap SNPs | Enrichment Folds |  | # overlap SNPs | Enrichment Folds |  | # overlap SNPs | Enrichment Folds |
| 1e-1 | 36,234 | 1.78 |  | 7,342 | 1.10 |  | 8,860 | 1.14 |
| 1e-2 | 10,211 | 2.97 |  | 946 | 1.26 |  | 1,295 | 1.17 |
| 1e-3 | 4,089 | 5.00 |  | 129 | 1.23 |  | 266 | 1.28 |
| 1e-4 | 1,777 | 6.22 |  | 56 | 2.31 |  | 85 | 1.37 |
| 1e-5 | 901 | 6.76 |  | 52 | 4.83 |  | 74 | 2.26 |
| 1e-6 | 402 | 5.33 |  | 51 | 5.89 |  | 72 | 3.29 |

RICHS placenta eSNPs below 10% FDR were used in the overlap analysis. For Birth Weight, Childhood Obesity and Childhood BMI GWAS, 16245523, 2439791, and 2496635 SNPs entered the analysis, respectively.
