# Supplementary material for: Genetic regulation of the placental transcriptome underlies birth weight and risk of childhood obesity
Source: PLoS Genet. 2018 Dec 31;14(12):e1007799. doi: 10.1371/journal.pgen.1007799 (PMC6329610; doi:10.1371/journal.pgen.1007799)
Supplement: S4 Table — (DOCX) [file pgen.1007799.s005.docx]

**Table. Comparing the eSNPs enrichment for GWAS signals of placenta vs. adult tissues**

|  | Two-sample Kolmogorov–Smirnov test pvalues (comparing to placenta) | | |
| --- | --- | --- | --- |
| **Tissue** | **BW** | **CO** | **CBMI** |
| AOR | <2.22E-16 | 5.53E-03 | 1.03E-04 |
| Blood | <2.22E-16 | 1.65E-06 | 2.93E-12 |
| MAM | <2.22E-16 | 1.16E-08 | <2.22E-16 |
| LIV | <2.22E-16 | 2.65E-12 | 4.92E-11 |
| SF | <2.22E-16 | 1.06E-03 | 7.01E-11 |
| VAF | 4.50E-06 | 7.03E-05 | 6.66E-16 |
| SKLM | <2.22E-16 | 2.93E-07 | <2.22E-16 |

Test are performed in the following procedure. We (1) identified the shared SNPs that are in the GWAS study (e.g. BW GWAS) and are also eSNPs (≤10% FDR) in the tissue of interest; (2) conducted LD pruning on the SNP list using a rather stringent threshold (ie, r^2^≤0.2), which capped the r^2^ among the pruned SNPs at 0.2; (3) performed two-sample Kolmogorov-Smirnov test (K-S test) between the GWAS pvalues of pruned placenta eSNPs and GWAS pvalues of pruned eSNPs of a given adulthood tissue.
