# Supplementary material for: Genetic regulation of the placental transcriptome underlies birth weight and risk of childhood obesity
Source: PLoS Genet. 2018 Dec 31;14(12):e1007799. doi: 10.1371/journal.pgen.1007799 (PMC6329610; doi:10.1371/journal.pgen.1007799)
Supplement: S5 Table — (DOCX) [file pgen.1007799.s006.docx]

| Table. Ethnicity distribution of the RICHSs placenta data set (n=150) | | |
| --- | --- | --- |
|  | **N** | **(%)** |
| Maternal Ethnicity (self-report) |  |  |
| White | *116* | *77.3* |
| Black | *10* | *6.3* |
| Other | *21* | *14.0* |
| Unknown | *3* | *2.0* |
